# OPTIM: a randomized phase II trial of nivolumab followed by nivolumab-ipilimumab or docetaxel at progression in recurrent/metastatic squamous cell carcinoma of the head and neck (OPTIM; AIO-KHT-0117)

**DOI:** 10.1038/s41416-026-03558-z

**Published:** 2026-07-18

**Authors:** Viktor Grünwald, Jürgen Alt, Mareike Tometten, Mathias Hänel, Philipp Ivanyi, Gunter Schuch, Konrad Klinghammer, Kerstin Gutsche, Justin Hasenkamp, Gunnar Hapke, Martin Mänz, Carolin Mogler, Dennis Hahn

**Affiliations:** 1 Carolus Institute of Urologic Oncology, University Hospital Essen, Essen, Germany; 2 III. Med. Klinik u. Poliklinik, University Hospital Mainz, Mainz, Germany; 3 Department of Hematology, Oncology, Hemostaseology and Stem Cell Transplantation, Medical Faculty, RWTH Aachen University, Aachen, Germany; 4 Internal Medicine, Hospital Chemnitz, Chemnitz, Germany; 5 Dept. Hematology, Hemostasis, Oncology & Stem Cell Transpl., Medical School Hanover, Hanover, Germany; 6 Hematology and Oncology Practice Altona, Hamburg, Germany; 7 Dept. Medical Oncology, Charite, Berlin, Germany; 8 Ambulatory Center for Hematology and Oncology, Cottbus, Germany; 9 Clinic for Hematology and Medical Oncology, University Medicine Goettingen, Goettingen, Germany; 10 Hematology and Oncology, Marien Hospital, Hamburg, Germany; 11 Corbin, Berlin, Germany; 12 Institute of Pathology, Techincal University Munich, Munich, Germany; 13 Hematology and Oncology Department, Hospital Stuttgart, Stuttgart, Germany

**Keywords:** Head and neck cancer, Tumour immunology, Outcomes research

## Abstract

**Background:**

Treatment options after PD-1 inhibition for recurrent/metastatic squamous cell carcinoma of the head and neck (R/M-SCCHN) remain limited. We investigated whether staggered immune checkpoint inhibition could improve outcomes compared with docetaxel in nivolumab-refractory disease.

**Methods:**

In this randomized phase II trial, adults with platinum-refractory R/M-SCCHN received nivolumab and were randomized at progression to nivolumab 3 mg/kg every 2 weeks plus ipilimumab 1 mg/kg every 6 weeks (NIVO-IPI) or docetaxel 75 mg/m² every 3 weeks (DOCE) until progression or intolerance. The primary endpoint was objective response rate (ORR) per RECIST 1.1; progression-free survival (PFS), overall survival (OS), and safety were secondary endpoints. PD-L1 expression was assessed in all patients.

**Results:**

Among 14 patients in the NIVO-IPI arm and 17 in the DOCE arm, ORR was 0% versus 17.6%, median PFS was 1.97 versus 3.66 months (*P* = 0.036), and median OS was 3.97 versus 11.9 months (*P* = 0.356), respectively. Twelve-month OS rates were 28.6% and 44.6%. Outcomes were similar irrespective of PD-L1 status. Treatment-emergent adverse events occurred in 84.6% versus 81.3% of patients, with grade ≥3 events in 38.5% and 68.8%.

**Conclusions:**

NIVO-IPI did not improve efficacy over docetaxel and showed numerically inferior survival, whereas docetaxel was associated with greater toxicity.

**Clinical trial registration:**

EudraCT Nr. 2017-003349-14

**Figure:**
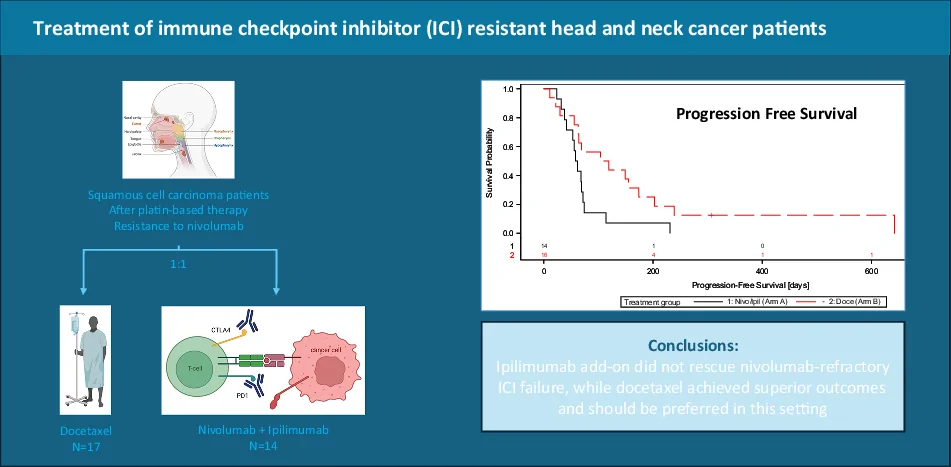

## Background

Squamous cell carcinoma of the head and neck (SCCHN) remains a significant global health burden, accounting for over 800,000 new cases annually [1]. The main causes for SCCHN are tobacco and alcohol consumption or human papilloma virus (HPV) infection, which influence prognosis [2–4].

In recurrent or metastatic SCCHN (R/M-SCCHN) chemotherapy results were poor [5]. The advent of immune checkpoint inhibitors (ICI) has improved outcomes, although five-year survival remains under 20% [6, 7].

ICI targeting the programmed cell death protein-1 (PD-1), such as nivolumab and pembrolizumab, have become a standard of care (SOC) in both first- and second-line treatment [8, 9]. More recently, two trials reported improved event or disease survival for the addition of PD-1 inhibitors to postoperative radio- or radio-chemotherapy, which indicates use of such agents in earlier stages [8, 9]. Despite these encouraging developments, a relevant proportion of patients do not respond or develop resistance to ICI treatment, creating a need for alternative strategies, which remain ill-defined.

The role of ICI that target cytotoxic T-lymphocyte-associated antigen 4 (CTLA-4) like ipilimumab is less understood in SCCHN. Dual checkpoint blockade, targeting both PD-1 and CTLA-4, has shown durable treatment effects in renal cell carcinoma and melanoma but also raised the risk of toxicity [10–12]. Therefore, ipilimumab was explored as a staggered escalated treatment approach in patients with inadequate response to PD-1 monotherapy, thereby reducing the number of patients exposed to dual CPI treatment. This sequential combination approach reported principle clinical activity in patients with urothelial and renal cell carcinoma [13–15].

However, its role in SCCHN has been less studied and resistance mechanisms to PD-1 ICI remain ill-defined [16]. Whether PD-1 refractory disease can be salvaged by the addition of a CTLA-4 inhibitor remains unknown. Docetaxel has principle activity in SCCHN and is commonly used in later lines of medical treatment. However, its toxicity profile and limited efficacy highlight the medical need for more effective and less toxic therapeutic alternatives [17, 18].

Our study investigated whether a staggered ICI combination of nivolumab + ipilimumab can improve outcomes for R/M-SCCHN patients resistant to PD-1 inhibitors and whether this combination is superior to conventional docetaxel. To evaluate this phenomenon, we focused on patients with early (prima) progression to nivolumab therapy in our trial. Patients with early progression ( ≤ 6 months) were considered ICI-refractory, mainly due to the atypical or delayed response, which may occur with ICI treatment [19].

## Methods

### Study design

We conducted an open-label, randomized, multi-center phase II clinical trial in Germany to compare the efficacy and safety of nivolumab (NIVO) in combination with ipilimumab (IPI) versus docetaxel (DOCE) in patients with R/M-SCCHN. The study enrolled adult patients with measurable disease who had previously received platinum-based chemotherapy. All patients received nivolumab (NIVO) monotherapy according to SmPC (240 mg q2wks) and patients who demonstrated progression within 6 months of start of NIVO treatment were eligible for randomization. Key inclusion criteria consisted of adults ( ≥ 18 years) with confirmed R/M-SCCHN or carcinoma of the nasal sinus, measurable disease (defined by RECIST v1.1), available tumor tissue, absence of active autoimmune disease and adequate organ function. Ethical approval (#7758AMGM2018) for the study was obtained and all patients provided written informed consent prior to participation (EudraCT Nr. 2017-003349-14).

### Randomization and treatment regimens

Patients were randomly 1:1 assigned to receive either nivolumab 3 mg/kg every two weeks (Q2W) combined with ipilimumab of 1 mg/kg every 6 weeks (Q6W) or docetaxel 75 mg/m^2^ q3wks. Treatment continued until disease progression, unacceptable toxicity, or withdrawal of consent. Randomization was stratified based on PD-L1 tumor expression (TPS ≥ 1%), time of progression on NIVO ( > 2 mo) and p16 status (OPC only). Prior to randomization a tumor biopsy was offered to patients to collect CPI refractory tissue.

### Tissue analysis

Specimens (formalin fixed paraffin embedded block or a minimum of 10 unstained slides) were send to central pathology (Institute for Pathology at the Technical University Munich) for quality control and immunohistochemistry (IHC) after Study inclusion (a minimum of 100 assessable cells per slide). PD-L1 staining utilized the 22C3 pharmDx antibody and membranous tumor proportion score (TPS) for analysis. TPS ≥ 1% was considered positive. In patients with OPC, P16 status was determined by IHC.

### Study endpoints

The primary endpoint was the objective response rate (ORR) according to RECIST 1.1 criteria, which was tested by χ^2^ test with 1-sided *α* = 0.05. Estimates reported 2-sided 95% exact confidence interval according to the Clopper-Pearson method. Secondary endpoints included Progression-Free Survival (PFS; defined as the time from randomization to the first documented progression of disease or death from any cause, whichever occurred first), Overall Survival (OS; Defined as the time from randomization to death from any cause), adverse events (AEs) recorded according to the Common Terminology Criteria for Adverse Events (CTCAE), version 4.03. Time to event distributions utilized Kaplan Meier methods.

### Sample size

A total of 154 patients was planned, based on a one-sided alpha of 0.05 and a power of 80% to detect a meaningful difference in ORR. The trial recruited from July 2018 until February 2020. The trial was terminated early in February 2020 due to changes in the treatment landscape for SCCHN patients. As a result, descriptive analyses were applied to evaluate outcomes.

### Patient and public involvement

Patients or members of the public were not prospectively involved in the design, conduct, reporting, or dissemination plans of this trial. Trial results were disseminated at a conference. Furthermore, the public will be informed upon publication of the trial results on social media platforms and results will be made accessible by open access publication.

## Results

**Fig. 1:**
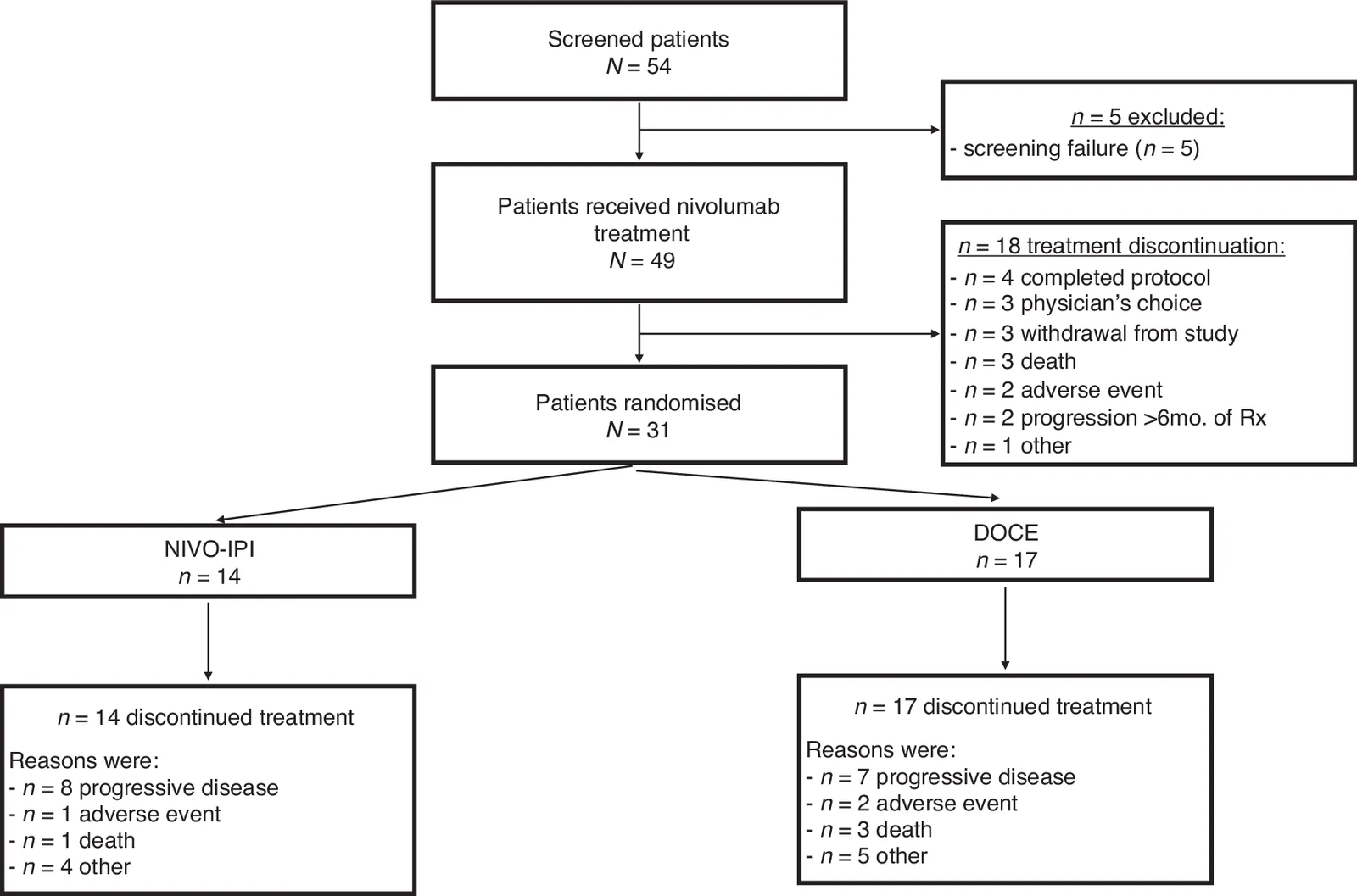
CONSORT diagram. Depicts numbers of participants who were randomly assigned and their treatment received

A total of 49 patients were enrolled into the study and received nivolumab monotherapy (Fig. 1) Early progression occurred in 31 patients, who were randomized to receive NIVO-IPI (*n* = 14) or DOCE (*n* = 17). The majority of the 31 randomized pts. had tobacco exposure (80.6%), P16-negative OPC (25.8%) and TPS positive disease (51.6%) (Table 1).

**Table 1:** Patients characteristics

	Nivolumab + Ipilimumab	Docetaxel	Total
	(*n* = 14)	(*n* = 17)	(*N* = 31)
**age**			
median (range), years	63.5 (51–84)	64.0 (39–81)	64.0 (39–84)
male, % (*n*)	64.3 (9)	76.5 (13)	71.0 (22)
Caucasian, % (*n*)	100.0 (14)	100.0 (14)	100.0 (14)
**Tobacco use**			
current or former, % (*n*)	78.6 (11)	82.4 (14)	80.6 (25)
never, % (*n*)	21.4 (3)	11.8 (2)	16.1 (5)
unknown, % (*n*)	0 (0)	5.9 (1)	3.2 (1)
median pack years (IQR)	25	35	30
	(3.0–40.0)	(7.0–50.0)	(4.0–50.0)
**ECOG performance status**			
0, % (*n*)	28.6 (4)	17.6 (3)	22.6 (7)
1, % (*n*)	71.4 (10)	82.4 (14)	77.4 (24)
**site of primary tumor**			
oropharynx, % (*n*)	35.7 (5)	41.2 (7)	38.7 (12)
oral cavity, % (*n*)	42.9 (6)	5.9 (1)	22.6 (7)
hypopharynx, % (*n*)	14.3 (2)	23.5 (4)	19.4 (6)
other, % (*n*)	7.1 (1)	29.4 (5)	18.8 (6)
**Relapse**			
local	35.7 (5)	58.8 (10)	48.4 (15)
metastatic	50.0 (7)	52.9 (9)	51.6 (16)
**metastatic sites**			
lung, % (*n*)	42.9 (6)	41.2 (7)	41.9 (13)
lymphnodes, % (*n*)	21.4 (3)	17.6 (3)	19.4 (6)
liver, % (*n*)	7.1 (1)	11.8 (2)	9.7 (3)
bone, % (*n*)	7.1 (1)	11.8 (2)	9.7 (3)
skin, % (*n*)	7.1 (1)	5.9 (1)	6.5 (2)
other, % (*n*)	7.1 (1)	5.9 (1)	6.5 (2)
**Marker**			
TPS ≥ 1, % (*n*)	57.1 (8)	47.1 (8)	51.6 (16)
TPS < 1, % (*n*)	42.9 (6)	52.9 (9)	48.4 (15)
oropharynx P16+, % (*n*)	20.0 (1)	16.7 (1)	18.2 (2)
oropharynx NE, % (*n*)	0	5.8 (1)	3.2 (1)
**previous therapies**			
cetuximab + platin, % (*n*)	42.9 (6)	17.6 (3)	29.0 (9)
radiochemotherapy, % (*n*)	57.1 (8)	76.5 (13)	67.7 (21)

### Treatment and exposure

The median number of cycles for NIVO monotherapy was 4 (range: 2–27). Randomized patients in the experimental arm received a median of 3.5 cycles of nivolumab (range 0–13) and 1.5 cycles of ipilimumab (range 0–5). Patients in the SOC arm received a median number of 5 cycles (range was 0–14) docetaxel.

### Objective response rate (ORR)

8 of 49 patients who received prior nivolumab monotherapy achieved an ORR (16.3%) (Supplementary Table. 1). 6 (12.2%) patients received prior nivolumab for more than 6 months and were not candidates for randomization. 4 of these 6 patients (66.7%) had an objective response.

Among randomized patients who received nivolumab-ipilimumab as a rescue strategy, the majority of patients achieved progressive disease or were not evaluable (NE) as best overall response (78.6%). None of these patients achieved an ORR (0%) and a minority had disease stabilization (SD; 21.4%). Patients who received docetaxel reported an ORR in 3 patients (17.6%) and SD in 29.4%, while 52.9% had either PD or were not evaluable (Table 2). The duration of response (DoR) was not reached (95%CI 28-not calculable) in patients with NIVO treatment. In randomized patients the frequency of responders was low and DoR was not calculable.

**Table 2:** Objective responses in all randomized patients.

	Nivolumab + Ipilimumab		Docetaxel	
	(*n* = 14)		(*n* = 17)	
	*n*	%	*n*	
CR	0	0	0	0
PR	0	0	3	17.6%
SD	3	21.4%	5	29.4%
PD	8	57.1%	3	17.6%
NE	3	21.4%	6	35.3%
ORR	0	0	3	17.6%

*NIVO-IPI* nivolumab + ipilimumab, *DOC* docetaxel, *CR* complete response, *PR* partial response, *SD* stable disease, *PD* progressive disease, *NE* not evaluable, *ORR* Objective response rate

### Progression free and overall survival

NIVO monotherapy achieved a median PFS of 0.95 mo (CI95% 0.92-1.81) (Supplementary Fig. 1) and OS of 16.3 months (95%CI 3.5-NR) (Supplementary Fig. 2). The 6 patients who were not randomized achieved a median PFS of 13.3 mo. (CI95% 0.79-NR) and OS of 28.0 mo. (95%CI 6.4-NR).

**Fig. 2:**
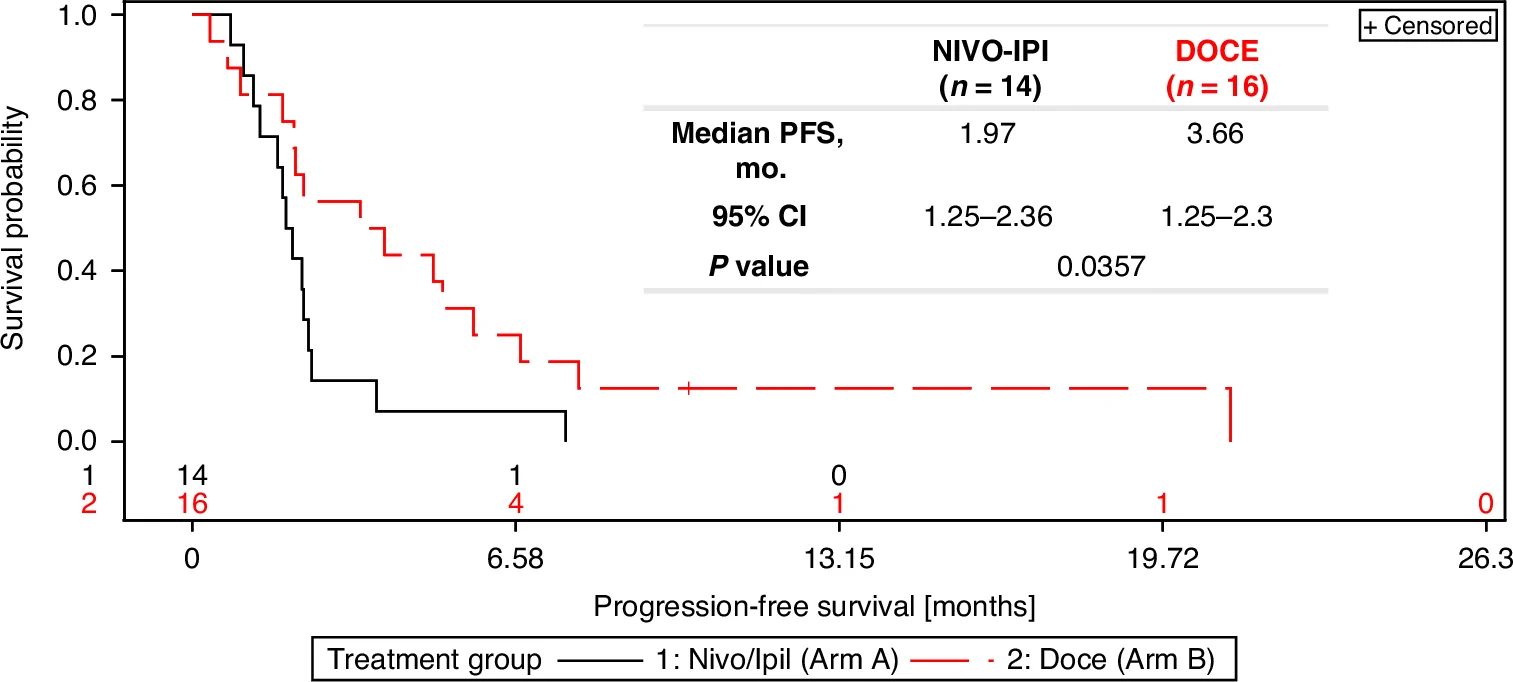
Median progression free survival in all randomized patients. NIVO-IPI: nivolumab + ipilimumab. DOC: docetaxel

NIVO-IPI was inferior to DOCE for progression free survival (PFS) (1.97 mo. (95%CI 1.25–2.36) vs. 3.66 mo. (95%CI 1.25–2.3; *P* = 0.036) (Fig. 2). The 6-months PFS rate (PFS_6mo_) was also in favor of DOCE (7.1% vs. 25.0%).

**Fig. 3:**
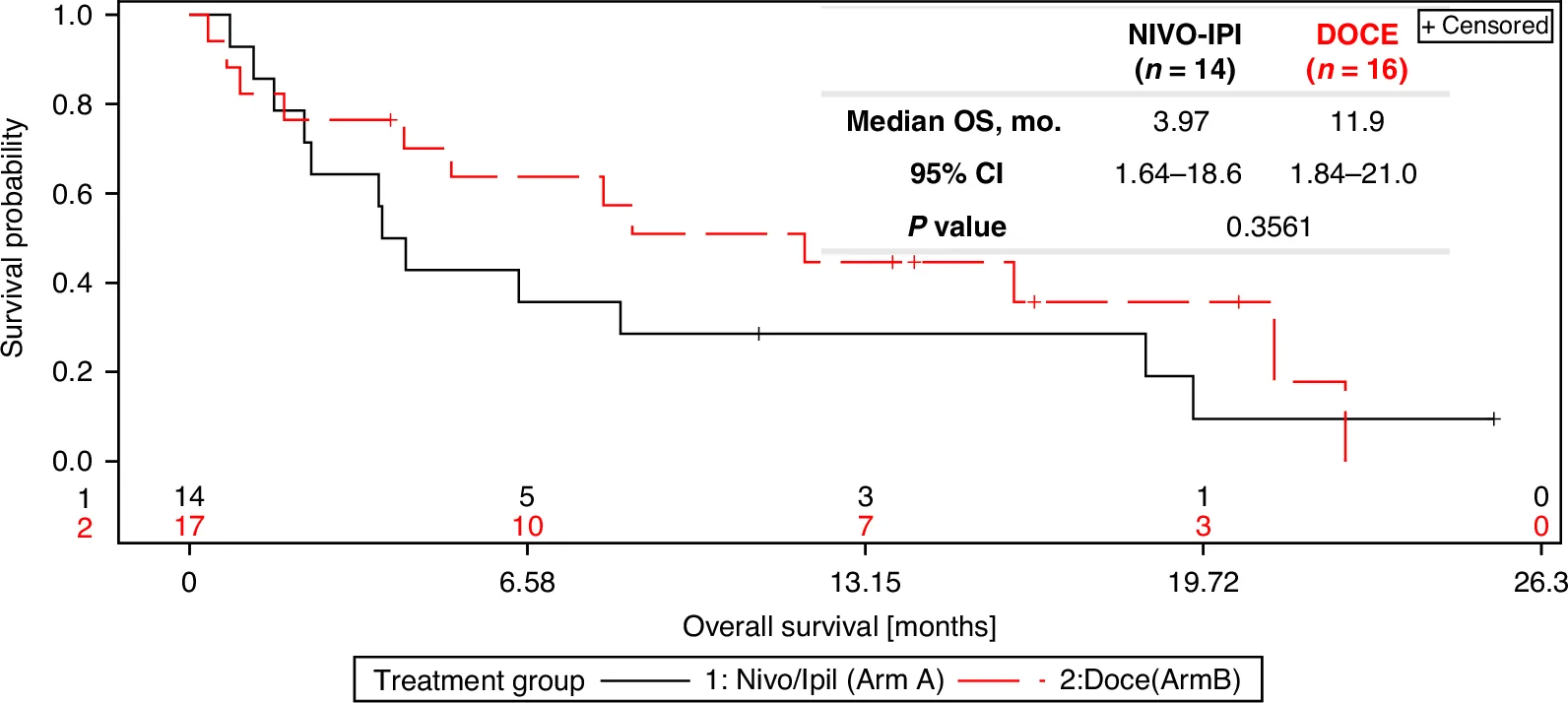
Median overall survival in all randomized patients. NIVO-IPI: nivolumab + ipilimumab. DOC: docetaxel

When NIVO-IPI and DOCE were compared for overall survival (OS), a trend for better outcome was reported for DOCE (3.97 mo. (95%CI 1.64–18.6) vs. 11.9 mo. (95%CI 1.84–21.0; *P* = 0.356), but did not reach significance (Fig. 3). This observation was further supported by the 12 months OS rate, which showed a trend in favor of DOCE (28.6% vs. 44.6%).

### Oncological outcome according to PD-L1 status

In 48 (97.9%) patients tumor specimens were sufficient and deemed assessable for further analysis. 28 (57.1%) patients had TPS ≥ 1%, which corresponded to 8 (57.1%) patients treated with NIVO-IPI and 8 (47.1%) with DOCE. In TPS ≥ 1% tumors, more patients experienced PD as best response to treatment with NIVO-IPI (35.7 vs. 17.6%). Interestingly, all 3 patients (17.6%) who achieved a PR with docetaxel treatment had TPS ≥ 1% (Supplementary Table 2).

The PD-L1 status (TPS < 1 vs. TPS ≥ 1%) did not impact PFS for NIVO-IPI (1.91 (95%CI 1.28–2.54) vs. 1.74 (0.83–2.65) or DOCE (3.42 (95%CI 0.87–5.97) vs. 2.27 (95%CI 0–6.14)). A similar finding was determined for OS (TPS < 1 vs. TPS ≥ 1%) for NIVO-IPI (3.75 (95%CI 0–8.60) vs. 3.68 (95%CI 0.95–6.42) or DOCE (21.11 (95%CI NC -NC) vs. 8.61 (95%CI 3.19–14.04)). Although numerical differences exist, the small sample size precludes strong conclusions.

### Safety

Most patients experienced a treatment emergent adverse event (TEAE) during the study. TEAE of any grade occurred in 47 (95.9%) patients treated with nivolumab (Supplementary Table 3), 11 (84.6%) patients treated with NIVO-IPI and 13 (81.3%) patients treated with docetaxel (Table 3). Grade ≥3 TEAE occurred in 24 (48.9%) patients with nivolumab, 5 (38.5%) patients with NIVO-IPI and 11 (68.8%) patients with docetaxel treatment. The most frequent TEAE on nivolumab treatment were fatigue (20.4%), tumor pain (12.2%), constipation (12.2%), hypothyroidism (12.2%) and weight loss (12.2%). In randomized patients, the most frequent TEAE with NIVO-IPI treatment were pain (30.8%), pneumonia (30.8%) and anaemia (23.1%) and with docetaxel treatment alopecia (37.5%), pain (25.0%), neuropathy (18.8%) and fatigue (18.8%).

**Table 3:** Treatment emergent adverse events (TEAE) with ≥ 10% incidence in all randomized patients

	Nivolumab + Ipilimumab				Docetaxel			
	*n* = 13				*n* = 16			
	any grade		grade ≥ 3		any grade		grade ≥ 3	
**Term**	***n***	**%**	***n***	**%**	***n***	**%**	***n***	**%**
**TEAE**	**11**	**84.6**	**5**	**38.5**	**13**	**81.3**	**11**	**68.8**
Pain	4	30.8	2	15.4	4	25	1	6.3
Pneumonia	4	30.8	2	15,4	1	6.3	1	6.3
Anaemia	3	23.1	2	15.4	2	12.5	0	0
Constipation	3	23.1	0	0	2	12.5	0	0
Fatigue	2	15.4	0	0	3	18.8	0	0
Infection	2	15.4	1	7.7	2	12.5	0	0
Oedema	2	15.4	0	0	1	6.3	0	0
Dyspnoea	2	15.4	0	0	1	6.3	0	0
Pleural effusion	2	15.4	0	0	1	6.3	0	0
Hypercalcaemia	2	15.4	1	7.7	0	0	0	0
Dizziness	1	7.7	0	0	2	12.5	0	0
Headache	1	7.7	0	0	2	12.5	0	0
Cough	1	7.7	0	0	2	12.5	0	0
Alopecia	0	0	0	0	6	37.5	0	0
Neuropathy	0	0	0	0	3	18.8	0	0
Stomatitis	0	0	0	0	2	12.5	0	0
Mucosal inflammation	0	0	0	0	2	12.5	0	0
Pyrexia	0	0	0	0	2	12.5	0	0
Conjunctivitis	0	0	0	0	2	12.5	0	0
Neutropenia	0	0	0	0	2	12.5	1	6.3
C-reactive protein increased	0	0	0	0	2	12.5	1	6.3
Leukopenia	0	0	0	0	2	12.5	1	6.3

Treatment related adverse events (TRAE) were reported in 17 (34.7%) and grade ≥3 in 3 (6.1%) patients treated with nivolumab monotherapy. TRAE led to temporary interruption of treatment in 8 (16.3%) patients and permanent discontinuation in 3 (6.1%). Severe TRAE occurred in 2 (4.1%) patients, which was fatal in 1 (2.0%: sudden death).

In randomized patients, TRAE were reported in 5 (38.5%) patients treated with NIVO-IPI, while 11 (68.8%) patients experienced TRAE on DOCE treatment. TRAE with grade ≥3 were reported in 2 (15.4) patients on NIVO-IPI and 6 (37.5%) on DOCE treatment. Temporary interruption of treatment due to TRAE occurred in NIVO-IPI vs. DOCE treatment in 2 (15.4%) and 5 (31.3%) patients, respectively. Permanent discontinuation due to TRAE was required in 1 (7.7%) and 3 (18.8%) with NIVO-IPI or DOCE treatment, respectively. Severe TRAE on NIVO-IPI or DOCE were reported in 1 (7.7%: diarrhea) and 4 (25.0%: asthenia, colitis, pneumonia, sepsis, syncope), respectively. Fatal severe TRAE was 0 (0%) and 1 (6.3%: sepsis) for NIVO-IPI and DOCE, respectively.

### Patient reported outcome

The low number of assessable questionnaires prohibited detailed exploration of PRO measures. An itemized list for PRO subdomain responses are depicted in the supplement. (Supplementary Table. 4)

10 (71.4%) patients in the NIVO-IPI arm and 13 (76.5%) patients in the docetaxel arm had PRO assessments at baseline and 10 (71.4%) and 9 (52.9%) patients at week 8, respectively. Baseline and week 8 mean global health scale (GHS) were 43.33 (SD: 19.17) and 39.17 (SD: 22.92) for patients with NIVO-IPI and 37.82 (SD: 22.72) and 46.30 (SD: 13.89) with docetaxel treatment, respectively.

EQ-5D visual analog scale (VAS) at baseline, week 4 and 8 was performed in 9 (64.3%) patients with NIVO-IPI treatment each and mean values were 43.6 (SD: 21.80) and 37.8 (SD: 18.39), respectively. 12 (70.6%) and 9 (52.9%) patients on DOCE treatment had baseline and week 8 assessments and means were 45.4 (SD: 26.41) and 53.1 (SD: 19.35), respectively.

## Discussion

The immune checkpoint inhibitors nivolumab and pembrolizumab are standard treatments in R/M-SCCHN patients following failure of platinum-based therapy [8, 9]. ICIs that target PD-L1 and CTLA-4 pathways have shown activity, justifying further investigation into their therapeutic potential for SCCHN patients [20, 21].

The advent of ICI therapies has created a data gap regarding subsequent therapies after ICI treatment in SCCHN patients. An important clinical question at that time was whether to commence sequential treatment with either ICI or chemotherapy. Retrospective studies indicate moderate efficacy of chemotherapy following ICI failure, with response rates and survival outcomes varying based on the type of subsequent chemotherapy [19, 22]. Taxanes are considered back-bone agents in the SCCHN therapeutic landscape [16], which putatively may enhance immune responses, potentially rendering it a preferred agent after ICI failure [23, 24].

A defining characteristic of ICI therapy is the durability of responses, which contrast sharply to the transient effects of chemotherapy [25]. Consequently, efforts to optimize ICI therapy have led to exploration of ICI combinations in various lines of therapy.

To mitigate the toxicity of combined ICIs, staggered ICI therapy was proposed, reserving combination therapy for cases unresponsive to single-agent ICI treatment and thereby limiting the exposure of patients to combined treatment. Furthermore, we included patients with R/M SCCHN after platinum failure into the study, who were treated with nivolumab until progression to allow rapid transition to randomization. The OPTIM trial is the first study to prospectively compare third-line therapies in PD-1 refractory SCCHN patients. Results indicated no improvement in progression-free survival or objective response rate with the addition of ipilimumab to nivolumab. In contrast, docetaxel demonstrated a significantly higher PFS and ORR, suggesting it may be a preferred option after ICI failure. However, the trial found only a trend for survival benefit from docetaxel, highlighting the need for more effective therapies. The early study termination led to a small sample size, which precluded detection of smaller differences and limited the exploration of subgroups. A major strength of our trial was the prospective centralized evaluation of PD-L1 in this academic trial. However, the stratification by PD-L1 status did not enhance for ICI-responder, which probably is a reflection of the utilization of archival specimens and the selection of ICI-refractory patient population in the OPTIM study.

Our observation is in line with the recently published PACEACE trial, which evaluated the combination of paclitaxel and cetuximab after ICI failure, demonstrating a high response rate of 47% after failure of PD-1 blockade [26]. Further, retrospective data support this observation with ORR of 39-53% for taxane-based therapies after ICI failure [27, 28].

While our study employed a distinct selection strategy and focused on sequential treatment in nivolumab-refractory disease, its findings align with the KESTREL, EAGLE or Checkmate 651 studies, which found no survival advantage with PD-(L)1 and CTLA4 ICI over chemotherapy in SCCHN [25, 29, 30]. This is consistent with the CONDOR trial, in which the addition of tremelimumab to durvalumab did not improve outcomes compared with durvalumab alone in previously treated SCCHN patients with low or absent PD-L1 expression [20]. These findings suggest that escalated ICI therapy may not be effective in PD-1 refractory SCCHN. The focus on ICI-refractory disease accentuated our results, and the lack of response and short PFS in this context suggested that staggered escalated ICI therapy should not be considered in SCCHN patients.

Meanwhile, the treatment landscape has evolved with the Keynote 048 study, which showed pembrolizumab, alone or with platinum-based chemotherapy, significantly improves survival in first-line SCCHN treatment [31]. Long-term follow-up indicates durable treatment effects from PD-1 inhibition [6]. Research continued into dual ICI treatments, as seen in the CM714 trial, which tested ipilimumab and nivolumab against nivolumab alone in first-line SCCHN [32]. Results did not show a benefit for the combination; ORRs were comparable, and no progression-free survival advantage was observed. Similarly, the CM651 and KRESTEL trials compared single or dual ICIs with cetuximab-based chemotherapy [29, 30]. Both trials found ICIs offered no survival benefit over chemotherapy, with ICI arms showing lower response rates and shorter PFS.

The therapeutic potential PD-1 ICI therapy has been further substantiated by recent data from two clinical trials, which incorporated ICIs into risk-adapted postoperative standard radiotherapy or chemo-radiotherapy regimens [10, 11]. The earlier use of ICIs will further fuel the discussion of the optimal sequence and putative value of switch to ICI, chemotherapy or the combination of both after failure of adjuvant ICI therapy.

These studies collectively underscore the challenges in treating ICI-refractory SCCHN and highlight the limitations of dual ICI strategies. The findings suggest a cautious approach to staggered ICI escalation in SCCHN patients, as evidence currently does not support improved outcomes in this setting.

## Conclusions

Immune checkpoint inhibitors such as nivolumab and pembrolizumab have become a valuable treatment option for patients with relapsed or metastatic squamous cell carcinoma of the head and neck. However, the optimal treatment strategy after ICI failure remains uncertain. Our study indicated that staggered or escalated ICI therapy does not appear to offer a substantial advantage in ICI-refractory R/M-SCCHN patients. Indeed, chemotherapy with docetaxel provided better outcomes than staggered ICI therapy with nivolumab and ipilimumab. Further studies are needed to refine post-ICI treatment protocols, focusing on improved patient outcomes.

## Supplementary information

sFig. 1: Median progression free survival in patients treated with nivolumab monotherapy

sFig. 2: Median overall survival in patients treated with nivolumab monotherapy.

sTab. 1: Objective responses in nivolumab (NIVO) treated patients.

sTab. 2: Objective responses in all randomized patients stratified by PD-L1 expression.

sTab. 3 TEAE NIVO Monotherapy

sTab. 4: Patient reported outcomes in all randomized patients.

## Data Availability

The lead author will consider written requests to share the data on a case-by-case basis.
