# Supplementary material for: OPTIM: a randomized phase II trial of nivolumab followed by nivolumab-ipilimumab or docetaxel at progression in recurrent/metastatic squamous cell carcinoma of the head and neck (OPTIM; AIO-KHT-0117)
Source: Br J Cancer. 2026 Jul 18;135(8):1247–54. doi: 10.1038/s41416-026-03558-z (PMC13642908; doi:10.1038/s41416-026-03558-z)
Supplement: Supplementary file 1 [file 41416_2026_3558_MOESM1_ESM.pdf]

sFig. 1: Median progression free survival in patients treated with nivolumab monotherapy.

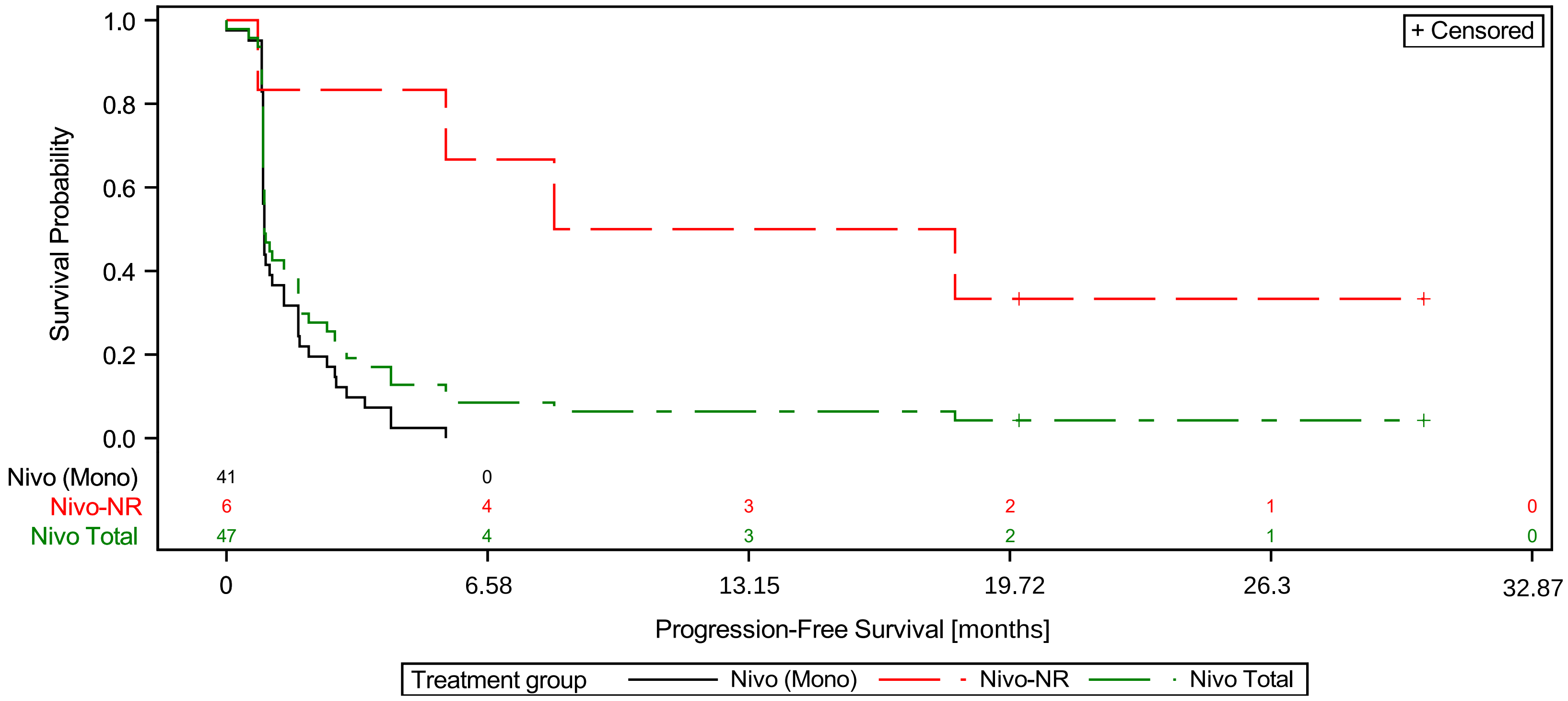

	NIVO	NIVO-NR	NIVO total
PFS (mo)	29<br>1.0	405<br>13.3	29<br>1.0
95% CI	(28-44)<br>(0.92-1.45)	(24-NR)<br>(0.79-NR)	(28-55)<br>(0.92-1.81)
