# Supplementary material for: OPTIM: a randomized phase II trial of nivolumab followed by nivolumab-ipilimumab or docetaxel at progression in recurrent/metastatic squamous cell carcinoma of the head and neck (OPTIM; AIO-KHT-0117)
Source: Br J Cancer. 2026 Jul 18;135(8):1247–54. doi: 10.1038/s41416-026-03558-z (PMC13642908; doi:10.1038/s41416-026-03558-z)
Supplement: Supplementary file 2 [file 41416_2026_3558_MOESM2_ESM.pdf]

sFig. 2: Median overall survival in patients treated with nivolumab monotherapy.

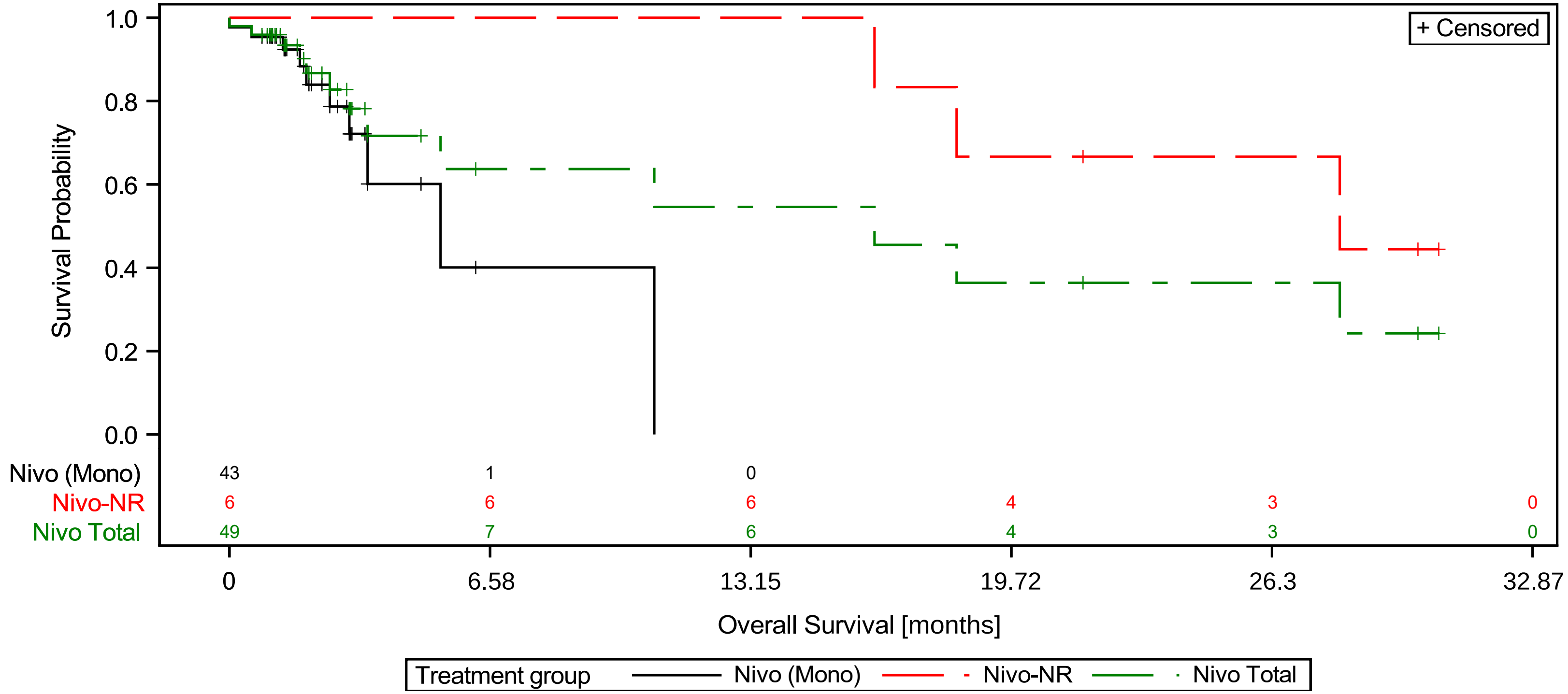

	NIVO	NIVO-NR	NIVO total
PFS	162	852	495
OS (mo)	5.3	28.0	16.3
95% CI	(92-NR)<br>(3.0-NR)	(495-NR)<br>(6.4-NR)	(106-NR)<br>(3.5-NR)
